# Excision of the Maxillary Bone

**Published:** 1855-01

**Authors:** 


### ARTICLE XIV.

### *Excision of the Maxillary Bone.*

A correspondent in the Medical Times, as the champion of Professor Syme, observes, in reply to a statement in that journal:

Mr. Syme, the object of the uncalled for criticism to which I allude, is represented by "Chirurgus" as "reiterating" a claim for "credit for being the first to perform the operation of removal of the superior maxillary bone in Europe."

This claim was never preferred by Mr. Syme. The credit he asks is that of "having happened to remove the first superior maxillary bone that was removed on this principle in Great Britain, and having entered upon the records of surgery, the first instance of this operation which they contain." I quote from a paper published by Mr. Syme, in the *Monthly Journal of Medical Science*, for January, 1854, and previously read by him to the Medico-Chirurgical Society of Edinburg. On the incorrect report of this sentence, given by your own Edinburgh correspondent, "Chirurgus" takes occasion to found his remarks, and does so, even although he was perfectly well acquainted with the true nature of the claim, which he states, indeed, in his postscript, in Mr. Syme's own words.

A faithful history of the operation will serve to show that Mr. Syme's claim is in every way a just one.

In his "System of Anatomical Plates," published in 1826, part 9, page 58, Mr. Lizars, of Edinburgh, first proposed in print, complete removal of the superior maxillary bone, when the seat of disease. Mr. Lizars attempted to perform the operation in December, 1827, but was prevented from accomplishing it by "the hæmorrhagic disposition of the gums and palate." M. Gensoul, of Lyons, apparently ignorant of Mr. Lizar's proposal, had already performed the operation in May, 1827; and he repeated it in January, 1828, and in March and April, 1829; as stated by "Chirurgus." These cases of M. Gensoul were not published, as "Chirurgus" himself owns, till 1833. In the interim, however, Mr. Syme had performed the operation on the 15th of May, 1829; and he published a full account of the operation in a paper in the *Edinburgh Medical and Surgical Journal*, of 1st July of that year; and further reported the progress of the case in the same journal of date 1st October, 1829. This was assuredly the first instance in which the operation was performed in Great Britain; and it was the case first put upon the records of surgery. A few months afterwards, Mr. Syme again performed the operation on a female patient, who presented herself at the hospital last autumn, in perfect health, twenty-four years having elapsed since the jaw was removed.

From misapprehension (?) of Mr. Syme's report of his first case, "Chirurgus" is led to imagine that Mr. Syme "had undertaken the operation without any hope of recovery from it." Now, Mr. Syme's statement is, that Dr. Ballingal and Mr. Nasmyth, agreed with him "in thinking that the case was a very fair one for practicing excision of the superior maxillary bone." Apprehensions were excited in the mind of the operator on finding, in the course of the operation, "that the morbid growth was not confined to the maxillary bone, but extended to the sphenoid in the base of the skull." The extent of the disease gave rise to an unfavorable prognosis, which was "not verified." That the disease did not re-appear in the base of the skull, Mr. Syme expresses himself as "at a loss to account for, unless the separation of a very large slough about a week after the operation, be considered a sufficient explanation." "Chirurgus" further "presumes that the operation was unsuccessful in establishing a cure." Presumption, indeed! in the face of Mr. Syme's report, published nearly three months after the operation, which he closes with these words :—"The man continues quite well, with the exception of a small gap in his cheek, and feels particularly comfortable in being freed from the distracting and incessant pain which formerly tormented him."

"It is necessary to premise," says, "Chirurgus," "notwithstanding Mr. Syme describes the disease as one of frequent occurrence, that the first, and so far as I have been able to discover, from the published report of surgical cases which he treated the only operation for its removal, undertaken by him, was performed in May, 1829." I know not how often Mr. Syme may have performed the operation since May, 1829; but this I do know, that the first operation which I saw Mr. Syme perform in the hospital here fully two years ago, was excision of the superior maxillary bone, and the operation has been repeated at least once since then, in the same theatre, by Dr. Mackenzie.

Seeing then, that Mr. Syme was the first to perform excision of the upper jaw, in Great Britain, and not Mr. Lizars, who proposed it; and, seeing that Mr. Syme was the first to record a case of it in the annals of surgery, it is not a little remarkable, that the writers of all the recent English text-books on surgery, Mr. South, in his learned translation of Chelius, Mr. Miller, Mr. Fergusson, Mr. Erichsen and Mr. Pirie, either should not have alluded to the matter at all, or if they have done so, should have awarded to Mr. Lizars, the credit which is due to Mr. Syme? The omission is the more reprehensible, as Mr. Syme published a detailed account of his case, (as already stated,) in a communication specially devoted to the subject, in the *Edinburgh Medical and Surgical Journal*, which cannot surely have escaped the observation of all his surgical brethren.

